# Cervicovaginal Microbiome: Physiology, Age-Related Changes, and Protective Role Against Human Papillomavirus Infection

**DOI:** 10.3390/jcm14051521

**Published:** 2025-02-24

**Authors:** Diana Alizhan, Talshyn Ukybassova, Gauri Bapayeva, Gulzhanat Aimagambetova, Kuralay Kongrtay, Nazira Kamzayeva, Milan Terzic

**Affiliations:** 1 School of Medicine, Nazarbayev University, Astana 010000, Kazakhstan; diana.alizhan@nu.edu.kz; 2 Clinical Academic Department of Women’s Health, CF “University Medical Center”, Astana 010000, Kazakhstankkongrtay@nu.edu.kz (K.K.); nazira.kamzaeva@umc.org.kz (N.K.); milan.terzic@nu.edu.kz (M.T.); 3 Department of Surgery, School of Medicine, Nazarbayev University, Astana 010000, Kazakhstan

**Keywords:** HPV, cervicovaginal microbiome, cervical cancer, *Lactobacillus*, local immunity, cervical dysplasia, cervical lesions, dysbiosis

## Abstract

**Background/Objectives:** Persistent high-risk human papillomavirus (HPV) infections are the leading cause of cervical cancer. Developing evidence suggests that the cervicovaginal microbiome plays a significant role in modulating HPV persistence and progression to cervical neoplasia. This review synthesizes the current knowledge on the interplay between the cervicovaginal microbiome and local immunity in HPV infections, emphasizing microbial diversity, immune responses, and potential therapeutic implications. **Methods:** A thorough review of the literature was performed using Embase, PubMed, Scopus, and Google Scholar, encompassing studies published between 2000 and 2024. Studies examining the composition of the microbiome, immune responses, and HPV-related outcomes were evaluated and synthesized into a comprehensive review. **Results:** A *Lactobacillus*-dominant microbiome, particularly with *L. crispatus*, creates a protective environment through lactic acid production, maintenance of low pH, and anti-inflammatory immune modulation, facilitating HPV clearance. Dysbiosis, often characterized by a dominance of *L. iners* and overgrowth of anaerobic bacteria, fosters chronic inflammation, cytokine imbalance, and a microenvironment conducive to HPV persistence and progression. Hormonal changes and menopause exacerbate these microbial shifts, increasing the risk of cervical lesions. Studies suggest that cytokine profiles and antimicrobial peptides significantly influence local immune responses, further modulating infection outcomes. **Conclusions:** The cervicovaginal microbiome is a critical determinant in HPV infection outcomes, with therapeutic potential for modulating the microbiome to enhance immune responses and prevent cervical cancer. Personalized microbiome-targeted therapies may offer a novel avenue for managing HPV and reducing cervical cancer incidence.

## 1. Introduction

Persistent high-risk human papillomavirus (HPV) infections cause up to 95% of precancerous cervical lesions and subsequent cervical cancer [1,2,3,4,5]. Up to 80–90% of women could be infected with either type of HPV at some point in their lifetime; however, the infection is usually cleared by the immune system in 12–24 months [1,2,6].

The local immune environment in the cervix and vagina, including the microbiome, could have an impact on HPV infection persistence [1,7]. In the cervicovaginal area, the optimal microbiome is featured by a high abundance of *Lactobacilli* species (spp.) [7]. In healthy conditions, the domination of *Lactobacilli* spp. in the cervicovaginal microbiome has been reported to support homeostasis through a variety of mechanisms, including maintenance of acidic pH serving as a protective factor against pathologic microbes [7]. Hence, the normal cervicovaginal microbiome influences the natural history of HPV infection and its protective role against other infections [1,7,8].

Although the development of precancerous cervical lesions and cervical cancer is associated with a long-term high-risk HPV infection persistence, the role of cervicovaginal microbiome stability in the natural history of cervical cancer is important [1,7,8]. Thus, in this review, we aim to summarize and update the knowledge on the role of the cervicovaginal microbiome in the pathogenesis and natural history of precancerous cervical lesions and cervical cancer. A better understanding of the local cervicovaginal immunity in the development of cervical malignancy might open new opportunities for the precancerous cervical lesion management and prevention of cervical cancer.

## 2. Material and Methods

**Figure 1:**
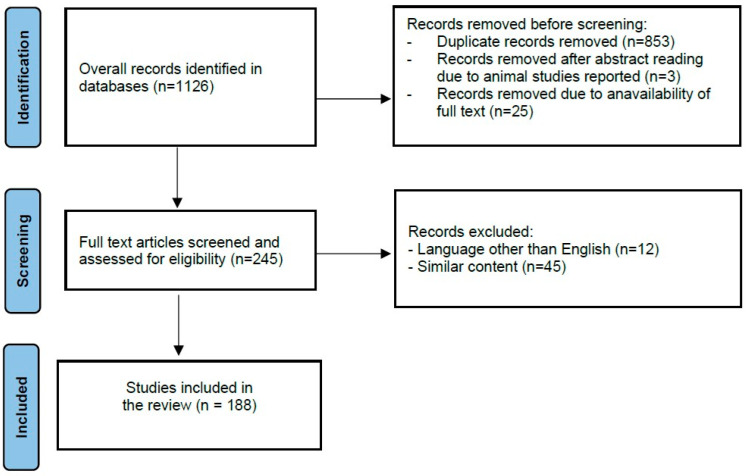
Literature search.

Articles published in English were searched in PubMed, Scopus, Embase, and Google Scholar databases from January 2000 to December 2024. The search was performed using the following keywords: “microbiota”, “cervical microbiome”, “vaginal microbiome”, “cervicovaginal microbiota”, “cervicovaginal immunity”, “*Lactobacillus*”, “human papillomavirus”, “HPV”, “high-risk HPV”, “host-microbial interactions”, “precancerous cervical lesions”, “cervical intraepithelial neoplasia”, “uterine cervical dysplasia”, and “early-stage cervical cancer”. Medical subject heading (MeSH) terms were used whenever available: “microbiota” (MeSH Unique ID: D064307), “*Lactobacillus*” (MeSH Unique ID: D007778), “uterine cervical dysplasia” (MeSH Unique ID: D002578), “Uterine Cervical Neoplasms” (MeSH Unique ID: D002583), “human papillomavirus” (MeSH Unique ID: D000094302), and “host-microbial interactions” (MeSH Unique ID: D000076662). The search was specified and targeted by using the abovementioned keywords and keyword combinations. The search results are presented in Figure 1.

The manuscript is best classified as a comprehensive review. This approach is particularly suited for synthesizing findings across diverse study designs and identifying overarching themes in a field that is still evolving. The comprehensive review structure allows for flexibility in integrating studies with varying methodologies and contexts, enabling the authors to provide a qualitative synthesis rather than a rigid, quantitative summary. This method is especially valuable when addressing complex topics like the interaction between the cervicovaginal microbiome, local immunity, and HPV, where the evidence base includes both experimental and observational studies. By adopting a narrative approach, the manuscript effectively highlights trends, identifies research gaps, and proposes potential areas for future exploration. Furthermore, this approach facilitates the incorporation of clinical and theoretical perspectives, bridging the gap between foundational research and practical applications. While it does not aim to map all available evidence comprehensively as a scoping review would, the narrative format allows for a nuanced discussion that aligns with this review’s objectives. The following research questions are discussed in this review:

1. What is the composition of a healthy cervicovaginal microbiome?
2. What are the age-related changes in the cervicovaginal microbiome?
3. Which microbial species and compositions significantly modify the HPV acquisition, persistence, clearance, and development of pre-cancerous/cancerous cervical lesions?
4. How does the cervicovaginal microbiome impact immunity against HPV?

## 3. Physiology of Cervicovaginal Microbiome

### 3.1. Microbiome Profile Within the Female Reproductive System

A wide range of microbial communities colonize the surfaces and cavities of the human body. These microorganisms coexist in a symbiotic relationship with the host, with the composition of each community shaped by the unique environmental and host factors at different body sites [9]. The makeup of these microbial populations varies across anatomical regions and could differ from one individual to another, as well as over time, due to complex interactions between the host and microbes, as well as changes in the environment [7,10].

The female reproductive tract acts as an essential microenvironment, where various microbial communities establish themselves and interact with the mucosal surfaces, creating a complex microbial ecosystem [11,12]. The vagina hosts a vast and complex microecosystem comprising billions of microorganisms [7]. Employing 16S rRNA gene sequencing has enabled the systematic identification of microbial biomass throughout the female reproductive tract [13]. Analysis of data from 110 individuals of reproductive age indicated that the vaginal environment harbors between 10^10^ and 10^11^ bacteria. Studies on the microecology of reproductive organs have demonstrated that the microbiome of the reproductive tract plays an essential role by influencing anatomical structure, tissue histology, and immune function [11]. Through its interactions with these components, this microbiome holds considerable potential in supporting reproductive health [11,14,15].

The microbiota composition in the female reproductive system is shaped by both host and environmental factors, which typically coexist congruently [16,17]. Changes in this microbiota can lead to physiological or pathological shifts within the reproductive tract. Aging, menstrual cycles, and estrogen fluctuations are key drivers of microbiota alterations [18]. Prolonged exposure to adverse factors may cause dysbiosis, contributing to reproductive tract diseases. Strategies aimed at restoring microbial balance hold promise for improving reproductive health [19,20,21,22].

Anatomically, the female reproductive tract is divided into the upper (uterus, fallopian tubes, and ovaries) and the lower (vagina and cervix) parts, which have direct exposure to the external environment [11]. Research on reproductive tract microbiota has revealed that it is not sterile but rather harbors diverse microbial communities [11,12,13,16,23]. Interestingly, despite their anatomical connection, distinct microbial differences exist, with a decrease in *Lactobacillus* and total bacterial load but increasing microbial diversity from the lower to the upper reproductive tract [11,12,16,21,23].

### 3.2. Healthy Vaginal and Cervical Microbiome

The vagina, which is an inlet part of the female reproductive tract, harbors a high diversity of bacterial biomass representing the resident vaginal microbiome [12,24,25]. *Lactobacillus* spp. are predominantly abundant at the genus level, comprising over 89% of the microbial population, whereas the presence of genera such as *Prevotella*, *Sneathia*, *Staphylococcus*, *Veillonella*, and *Streptococcus* remains a subject of ongoing debate [12,24,26]. Some researchers propose that a high prevalence of *Lactobacillus* within the vaginal microbiome signifies a healthy and typical microbial environment. The relative abundance of this dominant *Lactobacillus* spp. is thought to define various bacterial community structures, referred to as community state types [27,28,29]. Based on sequencing studies, five community state types were classified based on the vaginal microbiome: types I, II, III, and V have dominant *L. crispatus*, *L. gasseri*, *L. iners*, and *L. jensenii*, respectively, while type IV refers to the high diversity of the microbial ecosystem featured by obligate anaerobic bacteria [28,30]. Currently, community state types I, III, and IV are frequently observed in women and have been the focus of extensive research, while community state types II and V are relatively uncommon [31]. Research indicates that a vaginal microbiome predominantly composed of *L. crispatus* (type I) consistently supports vaginal health, whereas a microbial ecosystem with *L. iners* being dominant (type III) is associated with a higher susceptibility to vaginal infections [32,33]. Some microbial taxa within the vaginal microbiome can influence vaginal health and disease by modulating pro-inflammatory factors and their derivatives. This underscores the importance of thoroughly investigating the intricate relationships between these microbial communities and host inflammation responses [34,35,36].

There are variations in vaginal microbiome composition across different ethnic groups and races [13]. Out of the five community state types, type I, II, III, and V exist in 89.7% of white women and 80.2% of Asian women, while in black and Hispanic women, these indicators are lower—61.9% and 59.6%, respectively [13]. This diversity is possibly regulated by host genetic factors, such as the quantity and composition of vaginal mucus, epithelial cells, and immune system features [13]. Hereditary host features could play a more significant role in determining the vaginal microbiome among ethnic groups and races than behavioral and cultural factors [13,24,36,37,38,39]. For a long time, it has been commonly assumed that the cervical microbiome simply extends from the vaginal microbiome. However, emerging research has demonstrated that there are specific variations between the microbial communities of the cervix and the vagina [12]. Studies have shown that within the cervical microbiome, the phylum Firmicutes is the most prevalent, with the genus *Lactobacillus* making up a significant portion—up to 80.2% [40,41,42]. In addition, some studies have shown that lactic acid can be produced by *L. crispatus* in the cervix, which serves as an antimicrobial compound by inhibiting inflammation, therefore decreasing the incidence of local infections [43]. The phylum Bacteroidetes ranks as the second-most prevalent within the cervical microbiome, with *Prevotella* being the predominant genus, which is known as an essential member of the cervical microbiome [44]. *Lactobacillus* spp. thrive within the cervicovaginal anaerobic environment and synthesize a range of antimicrobial agents, including lactic acid, hydrogen peroxide, and bacteriocins [45,46,47,48]. These compounds play a crucial role in maintaining a balanced cervicovaginal microbiome and providing a protective barrier against pathogenic microorganisms. Specifically, *Lactobacillus* spp. are the primary producers of L-lactic and D-lactic acid, which help sustain the habitat’s pH below 4.5, while epithelial cells contribute approximately 20% of L-lactic acid production [46,47,48]. However, the role of hydrogen peroxide in the cervicovaginal microbiome is still debated. Although recent studies have shown its positive impact in inhibiting the overgrowth of pathogenic organisms [46,47,48,49], questions remain regarding the extent of its protective function and overall influence within the microbiome. Research by O’Hanlon et al. (2011) demonstrated that hydrogen peroxide, at physiological concentrations, exhibits minimal capability to eliminate pathogenic microbes [50]. At elevated levels, hydrogen peroxide displayed more pronounced antimicrobial activity but was found to be more detrimental to *Lactobacillus* spp. than to pathogenic organisms. These findings suggest that hydrogen peroxide may not play a central role as an antimicrobial agent in maintaining vaginal microbiome stability. Additionally, *Lactobacillus* spp. produce bacteriocins, antimicrobial peptides that can disrupt the cell membrane integrity of non-native microorganisms [51,52,53]. They also exhibit the ability to adhere to vaginal epithelial cells, thereby outcompeting other microbes for adhesion sites [53,54,55,56]. This adhesion is significant as it prevents pathogen attachment, a critical initial step in infection [56,57,58]. The dominant species of *Lactobacillus* ultimately influences the degree of protection offered by the vaginal ecosystem [59,60].

Local microbiome functional composition, along with its alterations in response to infection and disease progression, remains an area that requires further comprehensive investigation [59,60]. Key factors that influence the vaginal microenvironment, such as vaginal pH regulation, the role of lactic acid bacteria, microbial metabolites, and local inflammatory responses, collectively contribute to the maintenance of vaginal health [45,59,60,61]. Shifts in vaginal pH toward more alkaline conditions have been associated with an increased susceptibility to HPV infection [59,60,62,63,64]. Additionally, enzymes present in the vaginal ecosystem are pivotal in sustaining microbiota homeostasis and overall vaginal health. These insights into the factors that regulate vaginal microecology can be further explored to enhance prevention strategies and the management of precancerous cervical lesions [7,59,60].

## 4. Sex Hormone Levels and Cervicovaginal Microbiota

Variations in hormone levels from puberty to menopause also result in significant changes in the microbial ecology of the vagina [65]. The composition of the vaginal microbiota is primarily modulated by different levels of estrogen and progesterone [66]. Different concentrations of sex steroid hormones were found to affect the characteristics of the cervicovaginal microbiome during puberty, reproductive age, pregnancy, and menopause (Table 1) [65,66,67,68,69,70,71,72,73,74,75,76,77,78,79,80,81,82,83].

Evidence indicates that neonates harbor a diverse microbial community within the gut during the initial week of life, with ongoing, dynamic shifts in bacterial composition persisting until a relatively stable and mature state is attained between 1 and 3 years of age [70]. In the first week of life, the gut microbiota of healthy neonates is primarily composed of *Actinobacteria* (notably *Bifidobacterium*), *Proteobacteria*, *Bacteroides*, and *Firmicutes*, including *Lactobacillus*, which also predominates in the vaginal flora [67]. Studies suggest that microbial colonization in infants may originate at the maternal–fetal interface, with variations potentially influenced by the duration of gestation. However, the microecological features of the female reproductive system during these stages remain poorly understood, primarily due to the limited availability of samples collected from the reproductive tracts of infants and children [69,70,71].

Adolescence can be defined as a developmental period that encompasses progressive endocrine, reproductive, physical, and psychological maturation [75]. During early adolescence, when estrogen and progesterone levels remain low, the vaginal microbiota is characterized by high alpha diversity, including a predominance of *Aspergillus*, *Actinobacteria*, and various bacterial genera such as *Prevotella*, *Bacteroides*, *Gastrodia*, anaerobes, and small populations of *Bacteroides* and *Lactobacillus* [66]. Researchers explained these dynamics in the cervicovaginal microbiome with a rise in circulating estrogen levels only during the late stages of puberty, leading to increased glycogen production on and in epithelial cells lining the vagina, which serves to be a source for lactic-acid-producing *Lactobacilli* spp. that release hydrogen peroxidase and lactic acid to inhibit other bacterial species [75]. As gonadal maturation progresses, increasing sex hormone levels promote the thickening of the vaginal epithelium and the accumulation of glycogen, providing an essential nutrient source to support the growth and proliferation of vaginal microbes [72].

The reproductive years in women refer to the phase of life during which they are biologically capable of conceiving and bearing children. This period typically begins with menarche, the onset of menstruation, which usually occurs between the ages of 10 and 16, and continues until menopause. During menstruation, the vaginal microbiota undergoes significant changes, characterized by a shift in pH and microbial diversity. Its composition, similar to that in early adolescence, includes *Clostridium*, *Aspergillus*, *Actinobacteria*, and *Streptococcus*, which are unique to this phase [73,74,75,76]. The neutral pH of menstrual blood (7.2–7.4) reduces the antibacterial effects of lactic acid, promoting the growth of anaerobic microorganisms that utilize iron from menstrual blood as a nutrient source [74]. Microorganisms like *Streptococcus* and *Gardnerella* secrete iron-chelating complexes to facilitate growth, while neutrophil gelatinase-associated lipocalin (NGAL) in *Lactobacillus*-dominated ecosystems inhibits iron-dependent bacteria [75]. The elevated pH and iron levels, coupled with a decrease in *Lactobacillus*, enhance microbial diversity. As menstruation transitions to the follicular phase, rising sex hormones thicken the vaginal epithelium, increase glycogen secretion, and lower pH through lactic acid and hydrogen peroxide production, promoting *Lactobacillus* proliferation and reducing anaerobic bacterial diversity [76].

During pregnancy, specific immunological, metabolic, and endocrine changes influence the structural composition and abundance of microbial communities across the body, including the vaginal, intestinal, oral, and placental microbiota. Pregnancy is marked by increased diversity in vaginal microbes, including *Atopobium*, *Sneathia*, and *Gardnerella*, which decline by mid-pregnancy as *Lactobacillus* dominance increases, stabilizing the vaginal environment and lowering pH to limit pathological bacterial growth [77,78,79,80].

Women in menopause, typically around the age of 50, experience notable changes in sex steroid hormones, such as decreased estrogen levels and increased follicle-stimulating hormone levels. Upon reaching the menopause period, the level of estrogen hormone declines, thereby causing a reduction in *Lactobacillus* concentration and promoting the growth of anaerobic bacteria in the vaginal environment [84].

Gliniewicz et al. (2019) investigated the vaginal microbiome of perimenopausal women, considering hormone replacement therapy (HRT) [82]. They found that postmenopausal women on HRT had bacterial counts comparable to premenopausal women, while those not on HRT had bacterial counts nearly ten times lower (*p* < 0.05) [85]. Other studies have identified an increase in anaerobic and vaginosis-related bacteria, such as *Bacteroides mimics* and *Gardnerella vaginalis*, in postmenopausal women [83].

A study by Gandhi et al. (2020) analyzed patients where *L. iners* was the most abundant species followed by *L. gasseri* and *L. jensenii* with no detection of *L. crispatus* and *L. jensenii* in postmenopausal women [86]. Additional evidence comes from Yoshikata et al. (2022), who analyzed the vaginal microbiome of 70 Japanese women and demonstrated that three main *Lactobacillus* spp. (*L. crispatus*, *L. iners*, and *L. gasseri*) were the most dominant in premenopausal women, contributing to 71.6% of their vaginal microbiota [87]. Conversely, postmenopausal women had an almost negligible proportion of *L. crispatus,* with *L. iners* and *L. gasseri* becoming the main species, totaling 10.3%. The authors correlated that with the effect of declining estrogen [87].

## 5. Role of the Local Cervicovaginal Microbiome in HPV Infection and Its Persistence

### 5.1. Human Papillomavirus: Epidemiology, Persistence, and Cancerogenic Properties

Human papillomaviruses are small-sized, non-enveloped viruses that belong to the family of Papillomaviridae and possess a circular double-stranded DNA genome [6].

HPV particularly has a tropism for the transformation zone, the area between the paved stratified epithelium of the ectocervix and the cylindrical epithelium of the endocervix. This zone is “biologically” sensitive as it is the easiest path to reach the basal layer, which is the specific target of some carcinogenic agents like HPV [88].

HPV is proven to be a causative agent for 95% of cervical cancer cases. In 2020, 600,000 new cervical cancer cases were diagnosed in the world. It is most detected in females between 35 and 44 years old, with the average age being 50–53 [3,89,90,91,92]. The incidence rates of cervical cancer increase worldwide among women after the age of 25 years with the peak at the age of 50–54 years [92]. Cervical cancer remains the fourth leading cause of cancer-related death among women [3,89,90,91,92].

HPV types 16 and 18 were found in 75% of cervical cancer specimens, while HPV31, 33, 45, 52, and 58 were identified in an additional 20% of HPV-attributable cancers [3,90]. Although the development of cervical pre-invasive and invasive conditions is strongly linked with HPV infection, there are other factors involved [93]. Immunodeficiency state, smoking habits, *Chlamydia trachomatis* infection, and the patient’s age are among the other responsible factors that heavily predispose individuals to virus persistence. Imbalances in cervicovaginal microbiota and inflammation also predispose individuals to the persistence of HPV and further cancer progression [94,95].

Moreover, a high number of sexual partners, the simultaneous presence of other sexually transmitted diseases, the early start of sexual relationships, and poor hygiene practices also make the individual prone to developing infection [96,97,98].

According to recent research, there is a substantial correlation between immunological health and viral infection as well as the vaginal microbiota, which influences the female lower genital tract’s immune system [44,99,100].

### 5.2. Lactobacillus Species as a Defense Mechanism Against HPV

**Figure 2:**
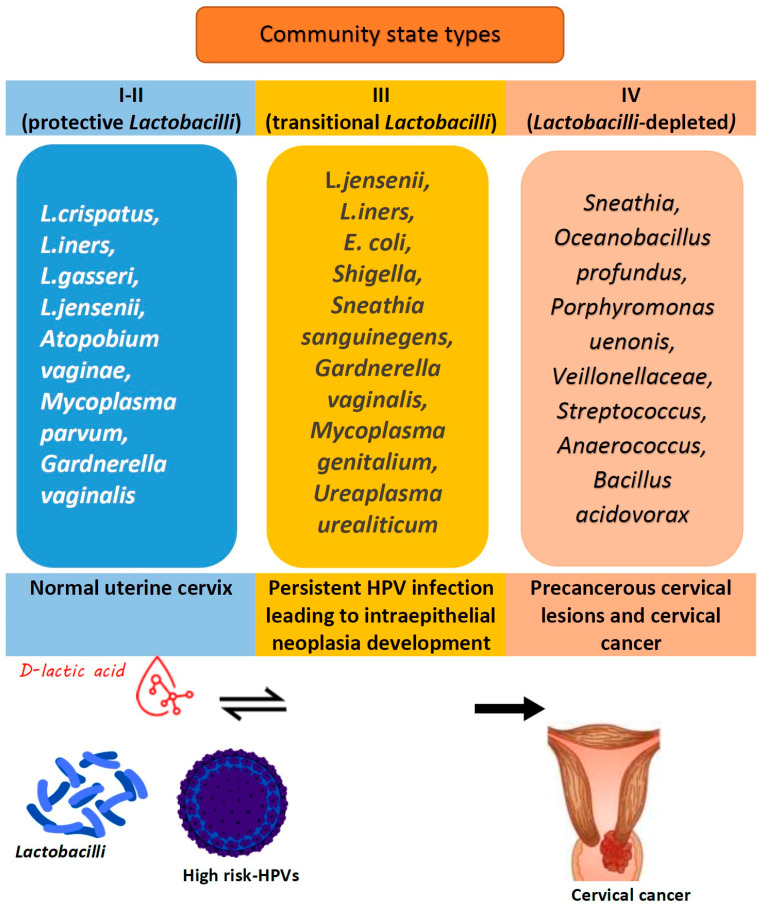
Association of cervicovaginal microbiota with HPV infection persistence. CIN—cervical intraepithelial neoplasia; CST—community state type.

In addition to the HPV infection properties (virulence, contagiousness, oncogenic potential, etc.), the vaginal microenvironment plays an intermediary role in making an individual susceptible to developing HPV persistence and further cancer (Figure 2).

The eubiotic microbiota in the vagina among reproductive-aged women mostly consists of various *Lactobacillus* spp., having protective reactions through the generation of multiple types of compounds such as lactic acid, bacteriocins, polysaccharides, peptidoglycans, and hydrogen peroxide, as well as through reducing the pH, enhancing the cervicovaginal mucus viscosity properties, and inhibiting the attachment of cells to epithelial tissue and HPV entry [101]. Specific strains of *Lactobacillus* residing in the cervicovaginal microbiota include *L. gasseri*, *L. crispatus*, *L. jensenii*, and *L. iners* [24,102].

It was shown that not all *Lactobacillus* spp. are defensive while maintaining the normal conditions in the reproductive tract of females and the stable microbial environment of the vagina [94].

#### 5.2.1. *Lactobacillus iners* and Cervicovaginal Microbiome

*L. iners* distinctively differs from *L. crispatus*, *L. gasseri*, and *L. jensenii* in several traits: high prevalence in vaginal microbiomes worldwide, smallest genome size, ability to produce only the L-isomer of lactic acid, and inconsistent correlations with the reproductive health of women [103]. This capability of producing only the L-lactic acid isomer makes it different from other major *Lactobacillus* spp., as *L. iners* lacks the gene encoding the enzyme D-lactate dehydrogenase [30]. Therefore, the L/D lactic acid ratio is highest in *L. iners*. Such a ratio may increase extracellular matrix metalloproteinase inducer (EMMPRIN), which in turn activates matrix metalloproteinase-8 (MMP-8), a metalloproteinase that breaks down the extracellular matrix, aiding bacteria in passing through the cervix and starting upper genital tract infections [104], which explains the reason why the overgrowth of *L. iners* in the vagina largely affects cervicovaginal microbiome disruption compared to other species. In addition to that, it was proven that D-lactic acid is more effective in the inhibition of bacteria in comparison to L-lactic acid [102], which serves as another reason for *L. iners’s* lesser capability in defending the cervicovaginal microbiome. One of the ways *Lactobacillus* spp. are thought to be able to stop anaerobic bacteria from colonizing the vagina is by producing hydrogen peroxidase. Apart from being limited in L-isomer lactic acid production, *L. iners* lacks the cellular and molecular resources necessary for pyruvate oxidation to produce hydrogen peroxidase [105].

According to Leizer et al. (2017), when an *L. iners*-dominated vaginal microbiome was compared against an *L. crispatus*-dominated microenvironment, more mediators were found to be released during microbiome disturbance in the presence of former species, which explains its larger role in defense mechanisms [106]. A higher level of NGAL and calprotectin production, both responsible for sequestering metal ions, released by bacteria [107,108] and MMP-8, serving as an indicator of disturbed vaginal ecology and further facilitating other defense pathways [109], are all the result of *L. iners* predominating the cervicovaginal microbiome under non-physiological conditions [106]. In addition to that, the studies postulated that there is enhanced generation of alpha-amylase as well to set a selective environment for *Lactobacillus* spp. besides *L. iners* utilizing glycogen mostly for their growth—another way to set homeostasis back. Moreover, *L. iners* obstructs dangerous bacteria from acquiring essential nutrients, such as iron, and impedes their persistent proliferation by activating the innate immune response in vaginal epithelial cells [110].

Kwak et al. (2020) were able to construct the first complete genome of *L. iners* and identify the high adaptability of this species to changing conditions. One of the causes of the abrupt decline in *Lactobacillus* spp. in cases of bacterial vaginosis was bacteriophages; however, *L. iners* can enhance defense mechanisms such as the type I restriction modification (RM) system, clustered regularly interspaced short palindromic repeats (CRISPRs), and its unique *hsdR* gene to resist bacteriophage invasion during bacterial vaginosis [111].

Leizer et al. (2017) reported that the *L. crispatus*-dominated cervicovaginal microbiome showed low levels of hsp70 and increased autophagy, while the reverse was true for the *L. iners*-dominated microbiome under stress conditions. Also, Toll-like receptors were activated for inducing pattern recognition receptors in the latter condition [106].

Vaginal microbiota characterized by non-*Lactobacilli* spp. or *L. iners* predominance correlated with up to five-fold greater odds of prevalent HPV and up to three times greater odds for high-risk HPV and dysplasia/cervical cancer when compared to an ecosystem with *L. crispatus* as the leading type [112].

This microorganism was determined to be the most dominant species in women with high-risk HPV infection in a study conducted in Singapore [113]. According to a systematic review performed in 2021, the role of *L. iners* was not exactly determined, as the review suggested a dual role of this microbe in maintaining a healthy vaginal environment while also outnumbering other species during bacterial vaginosis, largely contributing to a disturbed microenvironment [114].

A study which involved 800 African women proved that an *L. crispatus*-prevalent and less *L. iners*-dominated cervicovaginal microecology has a strong association with a lower chance of developing sexually transmitted infections [115]. Another study supports this finding and reported next-generation sequencing (NGS) results highlighting *L. iners*’s consistent prevalence in HPV-diagnosed as well as healthy patients [26].

Upon thorough investigation and comparison among persistent, transient, and healthy HPV cervicovaginal microbiome states, Qingqing et al. (2021) identified *L. iners* species to be correlated with the transient condition, with persistent infection having the lowest overall *Lactobacilli* count, though it was relatively abundant among other species [116].

#### 5.2.2. *Lactobacillus gasseri* and Cervicovaginal Microbiome

The addition of *Lactobacillus*, particularly *L. gasseri*, *L. fermentum*, and *L. plantarum*, to treatment with antimicrobial drugs was found to be effective and beneficial for the prevention of recurrent HPV infection [76]. *L. gasseri* and *L. crispatus* co-cultured with a normal cervical cell line exhibited minimal cytotoxicity after 24 h, yet effectively inhibited the proliferation of cervical tumor cells (HeLa). Interestingly, researchers hypothesized that non-lactate molecules were specifically responsible for the demonstrated antitumor activities that were safe for normal tissues residing in the cervix [117,118,119,120]. Vaginal lactobacilli have been shown to have cytotoxic effects on cervical tumor cells in vitro, not considering lactic acid and pH [118,119].

According to Nicolò et al. (2022), all species of vaginal microbiota stimulated the production of interferon-gamma (IFN-γ), interleukin-17 (IL), IL-6, and IL-10 in considerable amounts, with insignificant production of interferon-alfa (IFN-α), interferon gamma-induced protein 10, and IL-4. Among selected species of *Lactobacilli*, *L. gasseri* resulted in the highest amounts of IFN-γ and the lowest amounts of IL-17, whereas *L. iners* had optimally induced IFN-γ and IL-17 release [121,122].

Females with a cervicovaginal microbiota that was extremely diversified or dominated by *L. iners* were more likely to be HPV-positive in a 16-week longitudinal trial, whereas a microbiota that was dominated by *L. gasseri* was linked to the fastest remission rate from HPV infection [123]. A study by Nicolò et al. (2022) demonstrated the immunomodulatory potential of *L. gasseri*, which produced the most effective stimulus resulting in IFN-γ production by human mononuclear cells [121,122]. Other researchers performed luminex cytokine/chemokine panel analysis and were able to extract and highlight particularly *L. gasseri* LGV03 as an agent that was beneficial for HPV clearance through alterations in the host’s epithelial immune response [124]. The studies confirmed that *L. gasseri* is essential for local immune response [121,122,124]. However, a prior cross-sectional study that used quantitative PCR revealed that women with HPV had a greater prevalence of *Gardnerella vaginalis* and *L. gasseri* [125].

An original study led by Xiao et al. (2016), which compared dynamic changes occurring in the vaginal microbiota with different treatment methods for bacterial vaginosis, concluded that in most bacterial vaginosis-developed cases, *Lactobacillus* spp. reduced the recurrence rate of this state, though some showed relapse within a month [126]. Another extensive review performed on HPV persistence and microbial species came to the conclusion that species of *L. gasseri*, *L. jensenii,* and *L. crispatus* exert protective effects from HPV, while species of *Anaerococcus tetradius*, *Fusobacterium*, *Gardnerella vaginalis*, *Peptostreptococcus*, *Sneathia,* and *L. iners* in combination with other factors lead to a higher HPV rate and a lower HPV remission rate [127]. Meanwhile, *L. gasseri* was commonly found in HPV-infected patients in another study together with *Gardnerella vaginalis* [128]. To support the latter, the latest data collected among Iranian women diagnosed with bacterial vaginosis and infected with HPV showed a statistically significant correlation, with *L. gasseri* and *L. jensenii* colony counts being high [129]. Furthermore, it was demonstrated that decreases in HPV infection development risks are positively correlated with the presence of certain *Lactobacillus* groups, *L. gasseri* and *L. rhamnosus* [129].

Thus, *L. gasseri* and *L. iners* are not uniformly consistent in appearance and function during infection and healthy states. While *L. iners* is more often showcased as a “contributor” to a disturbed microbiome profile and also “predisposes” individuals to persistent HPV, consequently leading to cancer development, *L. gasseri* is more associated with prevalence in a normal cervicovaginal microbiome and stays dominant under the bacterial vaginosis state, when most *Lactobacillus* spp. are low in number.

A study led by Atassi et al. (2019) investigated mechanisms beyond the defense of the cervicovaginal microbiome by strains of *L. crispatus* and *L. gasseri*, eventually proving that most strains exert non-strain-specific protective properties, whereas a few still exhibited strain-specific antimicrobial activities [130]. All strains showed lactic acid-dependent killing of bacterial vaginosis-related microbes: *Prevotella bivia*, *Gardnerella vaginalis*, *and Escherichia coli* [130]. *L. crispatus* mostly exploits hydrogen peroxide-dependent killing by direct contact, which was demonstrated in the same study.

In a study involving Chinese women who had not been vaccinated previously, *L. gasseri* dominated among participants in the HPV-positive group [131]. A recently conducted systematic review of nine original studies from 2013 to 2021 determined that in cases when cancer cells were co-cultured with *Lactobacillus* strains, certain species such as *L. gasseri*, *L. crispatus*, and *L. casei* were able to result in HPV clearance in vitro [132].

#### 5.2.3. *Lactobacillus crispatus* and Its Role in Cervicovaginal Microbiome Modulation

The dominance of *L. crispatus* is responsible for the mucosal microenvironment’s high lactic acid production and protective protein release, both of which have been closely linked to a healthy vaginal microbiome [95]. According to Nicolò et al. (2023), out of all *Lactobacillus* spp., *L. crispatus* was proven the most effective in protecting against high-risk HPV [122]. One of the ways *L. crispatus* exerts its protective effect is through the synthesis of high amounts of D-lactic acid enhancing the viscosity properties of the cervicovaginal environment, resulting in the immobilization of viruses [133]. The NGS study results revealed that *L. crispatus*, *L. iners*, and *L. taiwanensis* were the most represented species among HPV-positive patients with low-grade squamous lesions (LSILs) and high-grade squamous lesions (HSILs) [26].

The *L. crispatus*-prevalent cervicovaginal microbiome had a statistically significant positive correlation with risk reduction for new HPV type acquisition or clearance of existing HPV types [134]. Moreover, Reimers et al. (2016) concluded that the clearance of HPV infection in the presence of *L. crispatus* or other *Lactobacillus* subgroups is carried out by different biological mechanisms (other than acid-base status). The researchers suggest a beneficial effect of *L. crispatus* on the burden of HPV in HIV-positive and HIV-negative women; thus, *L. crispatus* could contribute to the reduction in HPV infection and cervical disease [134].

As shown in a study by Ghanavati et al. (2020), a bacterial cocktail consisting of *L. crispatus*, *L. gasseri*, and *L. jensenii* reduced the expression of HPV oncogenic markers, particularly E6, E7, cyclin A, and cyclin-dependent kinase 2 (CDK2), thereby diminishing the probability of malignant transformation in cervical epithelial cells [135].

#### 5.2.4. *Lactobacillus jensenii* and Its Role in Cervicovaginal Microbiome

According to Nicolò et al. (2022), *L. iners* and *L. jensenii* were found to be responsible for the slight induction of viral E7 gene expression, while *L. crispatus* never induced the synthesis of viral genes or proteins when tested on SiHa cells [121]. A study involving young Korean women that had been previously vaccinated against HPV showed that not only bacterial vaginosis-related microbes but also *L. jensenii* and *L. iners* had a positive association with high-risk HPV cases and higher microbial diversity in the cervicovaginal microbiome [136]. Interestingly, in a study involving HPV-positive and HPV-negative pregnant women who were tested for cervical microbial composition, community state type IV with the prevalence of *L. jensenii* was found in HPV-positive pregnant patients [137].

Fang et al. (2022) found that *L. jensenii*, *L. crispatus*, and *L. helvetius* were highly prevalent in healthy individuals, which undermines their biomarker significance [138]. The authors concluded that *L. crispatus* and *L. jensenii* are potent defenders against high-risk HPV infections among all other *lactobacilli* representatives [138]. In a study by Hu et al. (2021), *L. casei* LH23 was proven to be effective in reducing the expression of E6/E7 genes, which are important causative factors for the emergence and development of cervical cancer [139]. Another recent study proposed *L. acidophilus* and *L. paracasei* as promising candidates for inducing apoptosis in cervical cancer cells (Table 2) [140].

#### 5.2.5. Mechanisms Underlying Defense by *Lactobacilli* spp.

After analyzing the effect of *L. crispatus* on cervical precancerous cells, Wan et al. (2023) found that this strain could prevent the proliferation and migration of infected cells with no significant effect on cell invasion [117].

A study by Lebeau et al. (2022) uncovered a novel viral immune evasion mechanism; namely, it showed that HPV inhibits basal and pro-inflammatory-induced host defense peptide expression [149].

Later, a systematic review on the implications and mechanisms of the antiviral effects of *Lactobacilli* spp. by Farahmandi et al. (2023) categorized the antiviral mechanisms into three groups: directly affecting the viruses, production/synthesis of specific antiviral compounds, or immune system activation against viruses [150]. According to this study, *Lactobacilli* spp. has significant antiviral effects.

A novel microorganism found in patients with cervical cancer is *Porphyromonas asaccharolytica*, which was able to activate genes responsible for inflammation. The basis for the following findings might be correlated with the results by Lithgow et al. (2022), which imply *Porphyromonas* spp. interaction with extracellular matrix proteases, leading to collagen degradation and subsequent disturbance of the coagulation system [151].

Moreover, type I interferons, such as IFN-α, IFN-β, and TLR3, are globally downregulated in HPV-positive patients with long-term HPV persistence, as reported by Gao et al. (2023) [124]. *L. gasseri* LGV03 helps maintain immune system vigilance against potential pathogens and reduces the inflammatory effects of persistent infection [124].

Cervicovaginal epithelial cells’ pro-inflammatory characteristics may be modulated by *Lactobacilli’s* high lactate synthesis and low short-chain fatty acid (SCFA) production [152]. Vaginal *Lactobacilli* have been shown to have cytotoxic effects on cervical tumor cells in vitro, not considering lactic acid and pH [118].

## 6. Local Cervicovaginal Immunity

### 6.1. Mechanisms and Factors Involved in Immune Response

The cervicovaginal immunological profile plays a crucial role in protecting the female reproductive tract from infections while maintaining a balanced microbiome. This immunity is characterized by a complex interplay of cellular and humoral responses, involving various immune cells, cytokines, and antimicrobial peptides [153].

The cervix consists of two immunologically distinct regions: the ectocervix, which harbors microorganisms, and the endocervix, considered “sterile”, separated by the cervical transformation zone [49,154]. The epithelial cells in both regions play a crucial role in immune defense, forming physical barriers through mechanisms like mucus secretion and tight junctions, and producing antimicrobial agents [155].

The mucus secreted by cervical epithelial cells serves as a protective barrier, containing antimicrobial proteins such as lysozyme and lactoferrin that neutralize potential microbial threats [156]. Additionally, cervical epithelial cells secrete various antimicrobial peptides, cytokines, and chemokines, enhancing the immune response. Notable secreted factors include β-defensins, secretory leukocyte protease inhibitors, and immunoglobulins (IgA and IgG), which are essential in preventing ascending infections to the upper reproductive tract [157].

These epithelial cells also express pattern recognition receptors (PRRs), which detect microbial pathogens and activate immune responses [158]. In the ectocervix and vagina, immune cells like T-cells, natural killer (NK) cells, and macrophages work in conjunction with epithelial cells to sustain local immunity. The concentration of these immune cells fluctuates with the menstrual cycle and pregnancy, reflecting an increase in immune activity during certain reproductive phases [159].

The immune environment of the vaginal mucosa interacts with the composition of the vaginal microbiome and regulates its function [160,161]. This complex interaction involves pro- and anti-inflammatory cytokines, antibodies, and epithelial and immune cells, as well as antimicrobial peptides [17,37,162,163]. Epithelial and immune cells, including dendritic cells in the vaginal mucus, play a dual role by detecting infections and maintaining balance with the VMB. These cells identify microbial components (antigens) through PRRs such as Toll-like receptors (TLRs), triggering the production of antimicrobial peptides and immunomodulatory cytokines/chemokines. Dendritic cells are also essential for linking the innate and adaptive immune responses, by presenting antigens to immune cells: macrophages, neutrophils, T and B cells, and NK cells [164,165].

### 6.2. Local Immune Response to HPV Infection

The innate and adaptive immune responses constitute the primary line of host defense at mucosal surfaces against infections like HPV [165]. The mucosal barrier present in the vagina plays a protective role as a result of co-interaction between epithelial cells, microorganisms, and the immune system [115,166].

The mucus is a gel-like layer that is primarily made up of mucins (MUCs), which are highly glycosylated mucous glycoproteins [44,167]. MUC5AC, MUC5B, and MUC6 mucins are secreted by cervical cells into the cervix. These mucins then flow into the vagina, where interaction with bacteria and vaginal epithelial cell products appear to form the cervicovaginal microbiome [168].

Furthermore, defense is provided through vaginal antimicrobial peptides (AMPs) which are responsible for not allowing foreign proteins to adhere to cells lining the genital tract [169]. Among them, the class of defensin molecules provides several ways of defense against the commonest microbial agents invading the vagina, which include HPV as well as the herpes simplex virus (HSV) and human immunodeficiency virus (HIV). Particularly, human β-defensin-2 (HBD-2) was highly expressed when the vaginal environment was abundant with bacterial species appearing during HPV infection, namely *Atopobium vaginae* and *Prevotella bivia* [170]. Other representatives of AMP are secretory leukocyte protease inhibitors, LL-37, and surfactant proteins A and D [164]. Just as defensins provide protection, surfactant proteins also exert inhibitory effects on viruses by binding to gp120 and CD4 expressed on host cells [171].

In addition, IgG and IgA prevent vaginal epithelial cell adherence and intake [170]. Moreover, these immunoglobulins play essential roles in neutralizing infectious agents and facilitating their clearance from the vaginal microenvironment [172].

Another protective mechanism is realized by PRRs, specifically DNA sensors involved in innate immunity reactions. Several PRRs act to recognize DNA upon HPV entry: IFN-γ-inducible protein 16 [173,174], TLR2, TLR4 [175], TLR 5, TLR 9 [176], and cyclic GMP-AMP synthase [177].

The innate immune response employs polymorphonuclear neutrophils and macrophages, which are implicated in oxidative bursts by reacting myeloperoxidase present in them with hydrogen peroxidase released by *Lactobacilli* [178]. NK and dendritic cells (DCs) are activated upon virus-like particles (VLPs) used in vaccines against HPV. Specifically, there is an upregulation of CD86 and human leucocyte antigen DR (HLA-DR), responsible for DC maturation; cell surface activation markers (CD69 and HLA-DR), responsible for the further activation of NK cells; increased production of IL-12p70; and an increase in IFN-γ secretion and cytotoxic activity by NK cells [179], which demonstrates the immune responsiveness of these cells against HPV.

Vaginal microbiome composition monitors the activation of different antigen-presenting cells (APCs) such as memory Th1, Th17, and T_reg_ lymphocytes [180], cervicovaginal Langerhans cells (cvLCs), CD14^−^ DCs, CD14^+^ DCs, and CD14^+^ macrophages [181]. When HPV-negative women and those with persistent infection were compared against those who succeeded in HPV clearance, the number of LCs was higher, while not much difference was observed in the number of CD4^+^ T-cells, mDCs, or monocytes [182].

Furthermore, Th17 and IL-17 were determined to be critical components of the immune response throughout disease progression; however, this was not true for high-risk HPV cases [183]. Conversely, IL-17 was previously shown to suppress immunity in HPV-associated cases [184].

According to a review performed by Passmore and Williamson (2016), Toll-like receptor 9 (TLR-9) stimulates the release of several cytokines and chemical mediators, namely tumor necrosis factor alpha (TNF-α), interleukin-8 (IL-8), macrophage inflammatory protein-3α (MIP-3α), monokine induced by gamma interferon (MIG), and interferon alpha (IFN-α). Despite TLR-9’s ability to recognize DNA pathogen-associated molecular patterns, HPV possesses E6 and E7 proteins, which interact with molecules within the TLR-9 intracellular signaling pathway, resulting in a reduction in the amount of IFN-α secreted [185]. In vitro studies proved that a cervicovaginal microbiome composed of more diverse bacterial species was characterized by higher levels of pro-inflammatory cytokine levels, such as IL-1α, IL-1β, and IL-8 [37]. In vivo studies by Thurman et al. (2015) confirmed the presence of IL-1β and TNF-α in high amounts when the vaginal microbiome was disturbed [186].

Interestingly, certain major histocompatibility complex I (MHC I) alleles predispose individuals to HPV persistence, a fact which gives another ground for thinking that the affinities of HPV antigens displayed by MHC molecules vary, and they might or might not be immunogenic enough to effectively prime immune cells specific to HPV in protected vs. at-risk individuals [187].

Other researchers reported that due to the long persistence of HPV and HPV detection at late stages of disease progression, the virus might employ strategies to evade the host immune response [188]. Another mechanism utilized by HPV is through reducing antigen synthesis while the virus is in its vegetative life stage, a process termed the passive immune evasion strategy [188]. As the name implies, a virus expresses an extremely low number of proteins during the early stages of its lifecycle, so very poor antigen presentation occurs [188].

In general, the local cervicovaginal immune reaction’s extent is determined by community state types in the female vaginal microbiome: while type IV results in the highest pro-inflammatory reaction, type I and type II have the lowest protective properties [164].

## 7. Strengths and Limitations

This manuscript demonstrates several notable strengths. It offers a comprehensive literature overview on the cervicovaginal microbiome, its physiological roles, age-related variations, and its interactions with HPV, providing a holistic view of the subject matter. The authors conducted a robust literature review, utilizing multiple databases such as PubMed, Scopus, Embase, and Google Scholar, ensuring a broad and current literature base. By focusing on studies from the past two decades, this manuscript maintains relevance and comprehensiveness. Its emphasis on the interaction between local immunity and the microbiota in HPV persistence and progression offers a fresh perspective, adding depth to the understanding of cervical cancer pathogenesis. The inclusion and exclusion criteria are clearly defined, ensuring the quality and relevance of the incorporated studies.

Despite these strengths, this manuscript also has notable limitations. As a narrative review, it lacks the statistical rigor of a quantitative meta-analysis, which could provide stronger evidence for the relationships discussed. The restriction to English-language articles introduces potential selection bias, possibly excluding valuable research in other languages. The absence of original experimental or clinical data limits its capacity to present novel findings. Variability in the designs, populations, and methodologies of the included studies introduces heterogeneity, which may affect the consistency of its conclusions. Many of the cited studies are cross-sectional, limiting the ability to infer causality between microbiota changes and HPV outcomes. Additionally, integrating interdisciplinary perspectives, such as epigenetics and behavioral science, could provide a more comprehensive understanding of the microbial ecosystem and immunity interactions. These limitations suggest that further studies on the effects of cervicovaginal microbiota and its impact on the persistence of HPV should be further investigated.

## 8. Conclusions

The findings of this comprehensive review highlight the critical role of the cervicovaginal microbiome and local immune responses in shaping the natural history of HPV infection and its progression to cervical cancer. Cervical cancer, a leading cause of cancer-related mortality among female, is primarily driven by persistent infections with high-risk HPV types, notably HPV-16 and HPV-18. However, the persistence and progression of HPV infection are not solely dependent on viral presence but are significantly influenced by the composition and stability of the cervicovaginal microbiome and the host’s immune response.

A healthy cervicovaginal microbiome, dominated by Lactobacillus species, particularly *Lactobacillus crispatus* and *Lactobacillus gasseri*, is associated with a protective microenvironment that suppresses HPV persistence. These beneficial bacteria maintain an acidic pH through the production of lactic acid, hydrogen peroxide, and bacteriocins, creating a hostile environment for pathogenic microorganisms and modulating local immune responses. In contrast, microbial dysbiosis—marked by a reduction in Lactobacillus dominance and an overgrowth of anaerobic bacteria such as *Gardnerella*, *Prevotella*, and *Sneathia*—is closely linked to chronic inflammation, epithelial barrier disruption, and increased HPV persistence. Notably, *Lactobacillus iners* presents a paradox, being prevalent in both healthy and dysbiotic states, suggesting its dual role as a potential transitional species in microbiome instability.

The local immune response is another key factor in HPV infection outcomes. Although immune protective mechanisms are present, HPV employs sophisticated immune evasion strategies, such as downregulating interferon responses and limiting antigen presentation, enabling its long-term persistence and progression toward malignancy.

Hormonal fluctuations, particularly variations in estrogen levels, further influence the cervicovaginal microbiome. During reproductive years, high estrogen levels promote a Lactobacillus-rich environment by increasing glycogen availability, which supports lactic acid production. However, menopause-associated estrogen decline disrupts this balance, leading to a rise in vaginal pH and microbial diversity, thereby increasing the risk of HPV persistence and cervical dysplasia.

These insights into the interplay between the cervicovaginal microbiome, immune responses, and HPV infection suggest several promising therapeutic avenues. Modulating the microbiome through probiotics, prebiotics, or microbial transplantation could potentially enhance HPV clearance and restore microbial balance. Additionally, immunomodulatory therapies targeting the local immune environment may offer innovative approaches to prevent the progression of HPV-related lesions and reduce the burden of cervical cancer.

Therefore, the cervicovaginal microbiome and local immunity play a pivotal role in determining the outcome of HPV infections. A well-balanced Lactobacillus-dominant microbiome acts as a natural defense against HPV persistence, while microbial dysbiosis and immune evasion strategies contribute to the development of cervical cancer. Future research should focus on elucidating these complex interactions further and exploring microbiome-targeted and immunotherapeutic strategies to improve HPV management and cervical cancer prevention. This integrated understanding offers a promising pathway for developing personalized, non-invasive interventions that could transform the landscape of cervical cancer prevention and treatment.

## Figures and Tables

**Table 1:** Female cervicovaginal microbiome composition depending on the age.

Age Group	Predominant Microbial Genera	Description of Changes	References
Infancy and childhood	*Staphylococcus*, *Streptococcus*, *Enterobacteriaceae*, *Corynebacterium*	Leads to the dominance of a wide range of aerobes and facultative anaerobes.	[66,67,68,69,70,71]
Adolescent	Transition towards *Lactobacillus* spp. dominance, including *L. crispatus*, *L. iners*; presence of *Streptococcus*, *Staphylococcus*, *Corynebacterium*	The vaginal pH of a young girl changes from birth until pre-puberty to become neutral or slightly alkaline, followed by a low abundance of lactobacilli.	[66,68,72]
Reproductive period	Predominance of *Lactobacillus* spp. (*L. crispatus*, *L. iners*, *L. gasseri*, *L. jensenii*); presence of *Gardnerella*, *Prevotella*, *Atopobium*	Depending on the phases of the menstrual cycle, the overall increase in estrogen level leads to the dominance of *Lactobacilli* spp. and lower vaginal pH.	[73,74,75,76]
Pregnancy	Increased abundance of *Lactobacillus* spp., *Bifidobacteriaceae*, particularly *L. crispatus*; reduced microbial diversity	Both estrogen and progesterone contribute to the increased dominance of lactobacilli during pregnancy, which stimulates glycogen accumulation in the vaginal epithelial cells favoring *Lactobacilli* spp. colonization.	[77,78,79,80]
Menopause	Decreased *Lactobacillus* spp.; increased prevalence of *Gardnerella*, *Atopobium*, *Prevotella*, *Mobiluncus*, *Streptococcus*, *Staphylococcus*	Hormonal changes during menopause lead to a decrease in *Lactobacillus* dominance, resulting in increased microbial diversity. A higher vaginal pH increases the risks of infections.	[68,73,81,82,83]

**Table 2:** Cervicovaginal microbiome species association with HPV infection [40,41,47,48,141,142,143,144,145,146,147,148].

Author	Country	Study Type	Sample Size	Participants’ Age (Years)	Test Technique Used	Findings	Reference
Campisciano et al., 2019	Italy	Cohort study	90 women	20–40	Species-specific multiplex genotyping assay	*Lactobacillus crispatus* increased in CST I while *Prevotella timonensis* and *Sneathia sanguinegens* increased in CST IV. An efficient viral clearance was observed only in women from CST I, dominated by *Lactobacillus crispatus.*	[144]
Chao et al., 2019	China	Cohort study	151 women	20–65	Sequencing barcoded 16S rDNA gene fragments (V4) on Illumina HiSeq2500	*Bacteroides plebeius*, *Acinetobacter lwoffii*, and *Prevotella buccae* were found significantly more frequently in HPV-positive women.	[141]
Onywera et al., 2019	South Africa	Retrospective cross-sectional study	62 women	Average 34.5	Bacterial 16S rRNA gene	*Lactobacillus*, *Gardnerella*, *Prevotella*, and *Sneathia* were the most predominant genera in the phyla Firmicutes, Actinobacteria, Bacteriodetes, and Fusobacteria, respectively.	[40,41]
Chen et al., 2020	China	Cohort study	229 women	25–69	Deep sequencing barcoded 16s rRNA ThinPrep cytology test, colposcopy examination	The highest microbial diversity was observed in cervical cancer patients when compared to other CIN/lesion-statused groups. HPV contributed to the reduction in the abundance of species of *Prevotella*, *Bacillus*, *Anaerococcus*, *Sneathia*, *Megasphaera*, *Streptococcus,* and *Anaerococcus*.	[143]
McKee et al., 2020	Appalachia, United States	Population study	308 women	21–39	Illumina MiSeq sequencing of 16S rRNA gene amplicons	Women who were determined to have abnormal cervical cytology or high-risk HPV possessed increased relative abundance of *G. vaginalis* and reduced relative abundance of *L. gasseri.*	[145]
Yang et al., 2020	China	Exploratory and validation cohort study	2251 women	25–50	Metagenome sequencing and HPV genotyping	*Lactobacillus,* followed by the *Gardnerella* genus, was highly dominant in both HPV-16-infected women and healthy groups.	[148]
Kang et al., 2021	South Korea	Cohort study	23 women, 4 groups: healthy individuals, patients with CIN 2, 3, and ICC	Average 47.4	Amplicon sequencing was performed using the Ion Torrent PGM	*Gardnerella* and *Prevotella* were abundant in the CIN group and only one genus was abundant in the healthy control group (*Lactobacillus*). *Gardnerella* and *Streptococcus* were the only microorganisms that differed significantly between each group.	[147]
Lin et al., 2022	China	Population-based cohort study	448 women	20–74	Sequencing the region of the bacterial 16S V4 rRNA gene	The proportion of *Gardnerella* and Prevotella were markedly increased in HPV (+) patients. *Gardnerella* and *Prevotella* are the most high-risk combination for the development of HPV (+) in women.	[146]
Liu et al., 2024	China	Prospective observational cohort study	802 women	Age was not reported	High-throughput 16S rRNA sequencing technology	Infected group exhibited a lower abundance of *Lactobacillus* and a significantly higher abundance of *Pseudomonas*, *Bifidobacterium*, *Limosilactobacillus*, *Peptostreptococcus*, *Gardnerella*, *Prevotella*, and *Dialister*.	[48]

CST—community state type; ICC—invasive cervical cancer; rRNA—ribosomal RNA; rDNA—ribosomal DNA.
